# MicroRNAs hsa-miR-99b, hsa-miR-330, hsa-miR-126 and hsa-miR-30c: Potential Diagnostic Biomarkers in Natural Killer (NK) Cells of Patients with Chronic Fatigue Syndrome (CFS)/ Myalgic Encephalomyelitis (ME)

**DOI:** 10.1371/journal.pone.0150904

**Published:** 2016-03-11

**Authors:** Robert D. Petty, Neil E. McCarthy, Rifca Le Dieu, Jonathan R. Kerr

**Affiliations:** 1 CFS Group, St George´s University of London, Cranmer Terrace, London, United Kingdom; 2 Centre for Haemato-Oncology, Bart’s cancer institute, Queen Mary University of London, London, United Kingdom; 3 Centre for Immunobiology, The Blizzard institute, Queen Mary University of London, London, United Kingdom; 4 Grupo de Salud Publica, Escuela de Medicine y Ciencias de la Salud, Universidad del Rosario, Quinta de Mutis, Bogotá 111221, Colombia; National Institutes of Health, UNITED STATES

## Abstract

**Background:**

Chronic Fatigue Syndrome (CFS/ME) is a complex multisystem disease of unknown aetiology which causes debilitating symptoms in up to 1% of the global population. Although a large cohort of genes have been shown to exhibit altered expression in CFS/ME patients, it is currently unknown whether microRNA (miRNA) molecules which regulate gene translation contribute to disease pathogenesis. We hypothesized that changes in microRNA expression in patient leukocytes contribute to CFS/ME pathology, and may therefore represent useful diagnostic biomarkers that can be detected in the peripheral blood of CFS/ME patients.

**Methods:**

miRNA expression in peripheral blood mononuclear cells (PBMC) from CFS/ME patients and healthy controls was analysed using the Ambion Bioarray V1. miRNA demonstrating differential expression were validated by qRT-PCR and then replicated in fractionated blood leukocyte subsets from an independent patient cohort. The CFS/ME associated miRNA identified by these experiments were then transfected into primary NK cells and gene expression analyses conducted to identify their gene targets.

**Results:**

Microarray analysis identified differential expression of 34 miRNA, all of which were up-regulated. Four of the 34 miRNA had confirmed expression changes by qRT-PCR. Fractionating PBMC samples by cell type from an independent patient cohort identified changes in miRNA expression in NK-cells, B-cells and monocytes with the most significant abnormalities occurring in NK cells. Transfecting primary NK cells with hsa-miR-99b or hsa-miR-330-3p, resulted in gene expression changes consistent with NK cell activation but diminished cytotoxicity, suggesting that defective NK cell function contributes to CFS/ME pathology.

**Conclusion:**

This study demonstrates altered microRNA expression in the peripheral blood mononuclear cells of CFS/ME patients, which are potential diagnostic biomarkers. The greatest degree of miRNA deregulation was identified in NK cells with targets consistent with cellular activation and altered effector function.

## Introduction

Chronic Fatigue Syndrome / Myalgic Encephalomyelitis (CFS/ME) is characterised by severe and debilitating fatigue lasting 6 months with associated muscular, infectious and neuropsychiatric symptoms. CFS/ME has a world prevalence of 0.4–1% with 240,000 UK and 800,000 US affected individuals [1, 2]. Currently there is no accepted diagnostic test or diagnostic biological marker for CFS/ME. The clinical diagnosis of CFS/ME is based on a process of exclusion culminating in the fulfilment of a set of clinical criteria after six months of illness [3–5]. Thus there is a real clinical need to identify a diagnostic biomarker in this disease. A number of studies have looked for biomarkers in CFS/ME in either the mRNA [6–8] or serum [9] of CFS/ME patients, with limited success. Ongoing research has focused on characterising immune defects in CFS/ME as onset of symptoms is frequently preceded by evidence of immune insult [10]. Alterations in populations and numbers of leucocytes in the peripheral blood of patients have been confirmed [11, 12]. Recent research has identified significant immune deregulation in CFS/ME with altered cytokine expression and reduction in the killing capacity of cytotoxic cells the most consistent findings [11, 13, 14]. Changes in mRNA expression in the peripheral blood have been well documented in CFS/ME, with a predominant bias towards genes involved in: immune function, apoptosis, transcription, translation and virus infection [7, 8, 15–26]. mRNA gene expression studies have also been successful in defining subtypes of CFS/ME, each with a distinct symptom profile [24]. One group of molecules that have a role in regulating the translation of mRNA and have yet to be systematically surveyed in blood cells of CFS/ME subjects are microRNA (miRNA).

miRNA are a group of small non-coding RNA (~23nt) which function to regulate the translation of mRNA post transcription via direct degradation of mRNA transcripts or repression of translation. miRNA are crucial for maintaining normal haematopoiesis and moderating immune signalling cascades [27, 28] suggesting their utility as biomarkers in this disease.

In this study, we screened peripheral blood samples derived from patients with an established clinical diagnosis of CFS/ME compared to age matched healthy controls and identified deregulation of miRNA with functions consistent with published mRNA expression changes in CFS/ME. To determine in which cell type these changes in miRNA expression were occurring, deregulation was confirmed in an independent patient cohort of fractionated peripheral blood cells. Expression of hsa-miR-99b and hsa-miR-330-3p in natural killer cells exhibited the greatest degree of over expression. When characterised in NK cells, these miRNA targeted genes involved in activation, effector function, actin cytoskeleton and motility. This work supports and expands the current concepts of immune deregulation in CFS/ME and identifies putative biomarkers for disease diagnosis.

## Results

### Thirty-four miRNA demonstrate increased expression in CFS/ME peripheral blood

**Fig 1:**
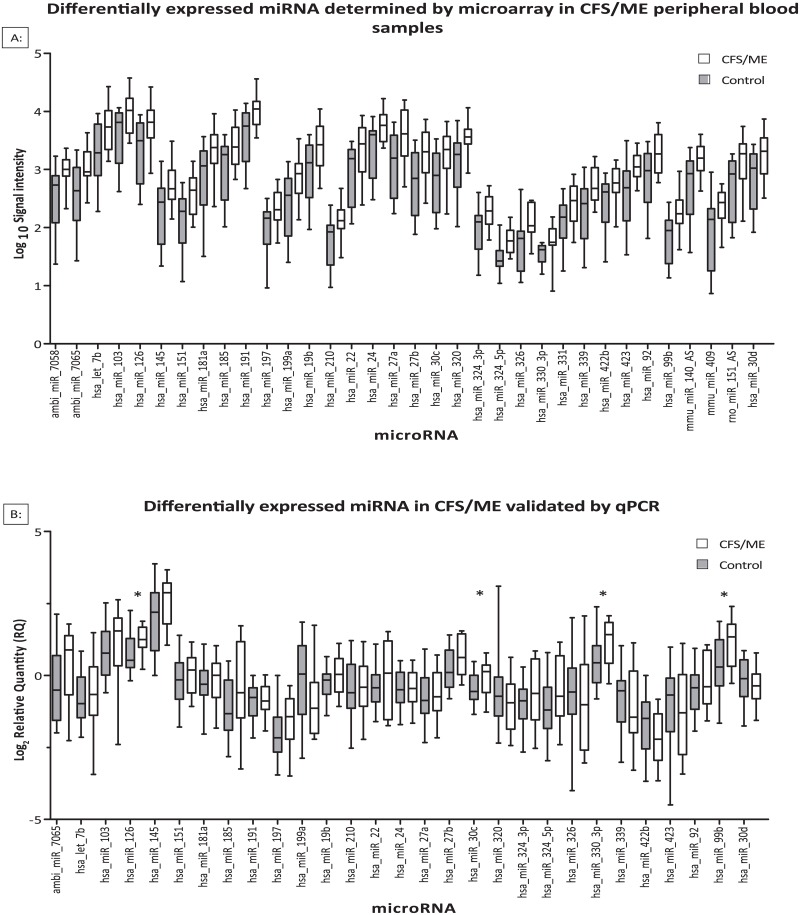
Statistically significant differentially expressed miRNA in CFS/ME analysed by microarray (A), validation by qPCR (B) boxes display interquartile range (25%-75%), error bars indicate minimum and maximum values *P>0.05.

To identify miRNA differentially expressed in CFS/ME, RNA was extracted from peripheral blood mononuclear cell (PBMC) samples and screened using Ambion Bioarray microarrays (version 1 targeting 385 miRNA sequences). Fifteen CFS/ME subjects meeting the Fukuda diagnostic criteria (N = 15) and age and sex matched controls (N = 30) were analysed (cohort characteristics summarized in S1 Table). A class comparison identified thirty four miRNA exhibiting differential expression greater than 1.5 fold change [false discovery rate (FDR) 0.05] (Fig 1 panel A, Full list S2 Table).

**Fig 2:**
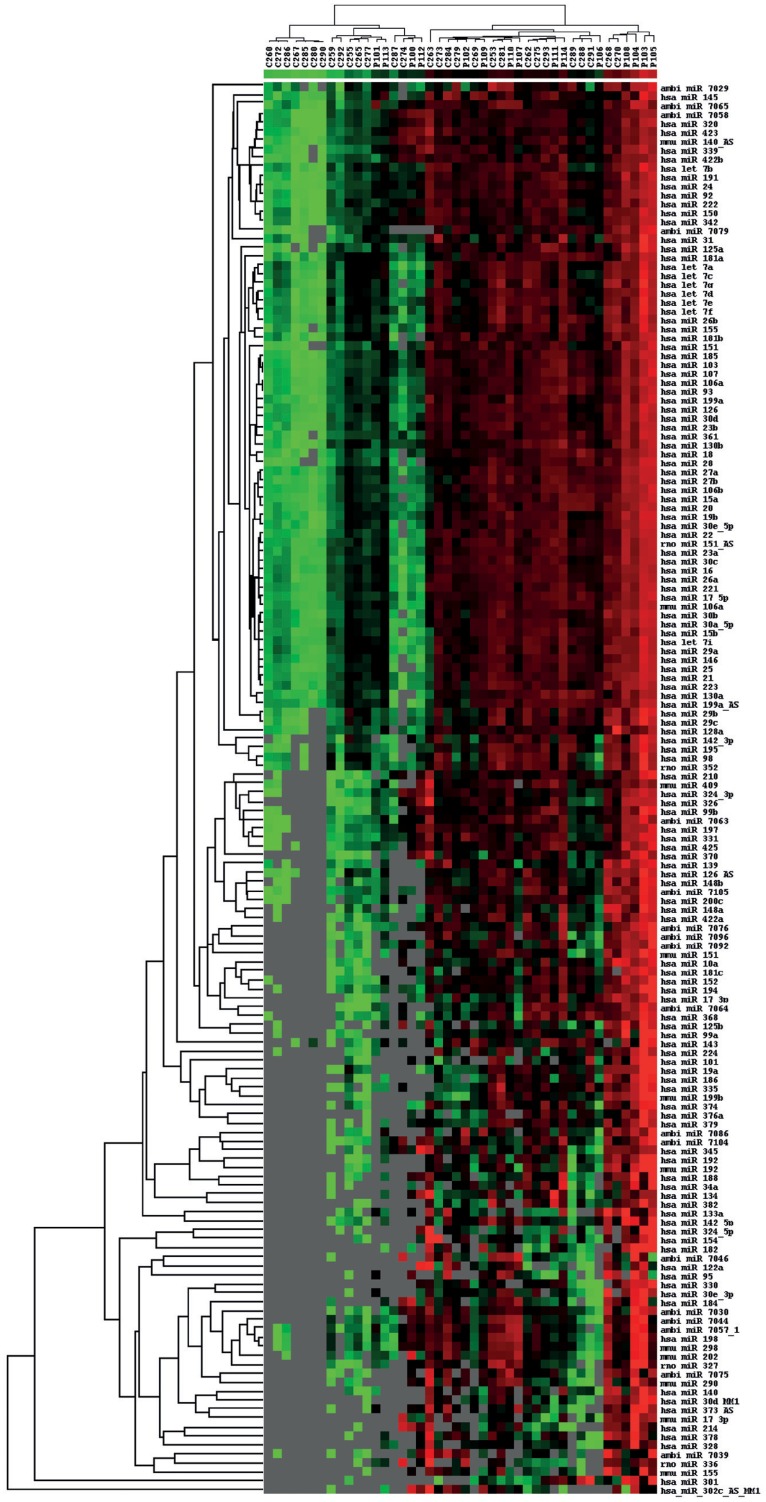
Unsupervised cluster analysis of miRNA microarray data. Clustering normalised miRNA expression values, present in ≥50% of samples by gene and sample, data is correlated by Euclidean distance using an average linkage to define the linkage tree (Performed using cluster V3.0 visualised in tree view). Each column represents one sample, each row a miRNA. miRNA expression is represented in red for high expression, green for low expression and grey for data excluded at normalisation.

Unsupervised cluster analysis (Fig 2) of the microarray data grouped the samples into two clusters. This analysis failed to fully resolve the CFS/ME and control samples into separate clusters. Approximately half the control samples (N = 14) group with four CFS/ME samples with low global miRNA expression. The remaining controls clustered with the majority of CFS/ME subjects demonstrating higher miRNA expression across the majority of miRNA analysed. The level of homogeneity between samples is high with an average correlation coefficient of 0.922 (+/- 0.068 SD) which may contribute to the incomplete resolution of the samples by clustering.

Interrogating this data by miRNA demonstrated the robustness of the expression data with miRNA known to be expressed in clusters grouped by expression in the analysis. This includes the let-7 miRNA cluster consisting of eight related miRNA [29] and miR-103 and 107 which have related functions and similar expression patterns [30]. We looked for correlation between miRNA gene expression data and clinical questionnaire scores from five general health questionnaires, however, none was found. This data demonstrates that miRNA were deregulated in the peripheral blood of CFS/ME patients and that levels of these miRNA were independent of symptom severity.

### Significant up regulation of microRNAs hsa-miR-99b, hsa-miR-330-3p, hsa-miR-126 and hsa-miR-30c in CFS/ME blood samples

The miRNA expression changes of 29 out of the 34 miRNA identified in the array screen were validated in the same samples by Taqman quantitative PCR. Five miRNA (ambi-miR-7058, hsa-miR-331, mmu-miR-140-AS, mmu-miR-409, rno-miR-151-AS) were not included as Taqman assays were unavailable. Four of the 29 miRNA (hsa-miR-99b, hsa-miR-330-3p, hsa-miR-30c & hsa-miR-126) exhibited significant differential expression ≥ 1.5 fold between the CFS and control cohorts at P≤0.05, using an additional statistical exclusion filter rejecting datasets if the 95% confidence interval for the fold change ratio included one (i.e. the probability of no change) (Fig 1 Panel B).

### Four miRNA have potential to be effective biomarkers in CFS/ME by ROC analysis

**Fig 3:**
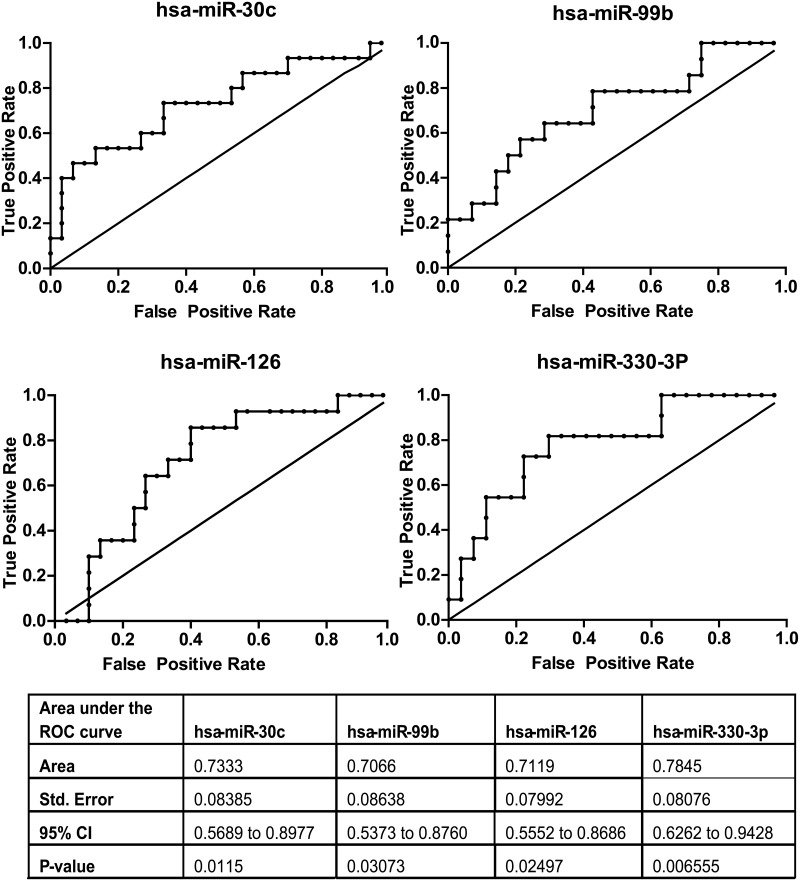
Receiver operating curves of the four differentially expressed miRNA with confirmed expression changes after qRT-PCR analysis.

To identify if the four confirmed differentially expressed miRNA (hsa-miR-99b, hsa-miR-330-3p, hsa-miR-126, hsa-miR-30c) could act as biomarkers for CFS/ME diagnosis, Receiver Operating Characteristics (ROC) were carried out for each set of RQ values for each miRNA. This statistic describes the ability of a variable to classify any sample into two groups and has previously been used to define serum biomarkers in CFS/ME [9, 31]. The four miRNA tested had area under the curve (AUC) values ranging from 0.71 to 0.78. All four miRNA tested exhibited statistically significant differences in AUC compared to a hypothesis of random chance (AUC = 0.5). Hsa-miR-330-3p was identified by this analysis as the mostly likely variable to differentiate CFS/ME patients from controls, demonstrating the lowest P-value and the greatest AUC (Fig 3). These data demonstrates that within the confines of this study these four miRNA were diagnostic biomarkers of CFS/ME.

### Hsa-miR-99b is significantly up regulated in NK cells in CFS/ME patients

To examine whether the miRNA changes observed in whole blood samples were specific to a particular PBMC population, 20 patients and 20 age and sex matched healthy volunteers were recruited using the same clinical diagnostic criteria as in the previous study. Blood samples were taken and PBMCs isolated. Constituent cells were fractionated using sequential rounds of positive selection using CD14+ (monocytes), CD19+ (B-cells), CD56+ (NK cells) and CD3+ (T-cells) microbeads. Flow cytometry of the fractionated populations demonstrated an average purity of 96.9% (+/- 2.1% SD) for monocytes, 88.9% (+/- 7.6% SD) for NK cells, 84.4% (+/- 8.8% SD) for B-cells and 91.2% (+/- 5.3% SD) for T-cells. Absolute cell counts post fractionation failed to show statistically significant differences between population groups for the four leukocyte subsets interrogated (data not shown).

**Fig 4:**
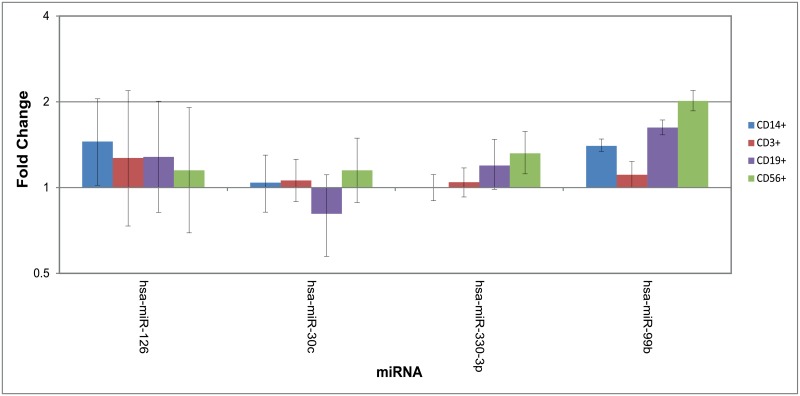
Four miRNA demonstrating differential expression by microarray and qRT-PCR tested by qRT-PCR in four cell populations. bars highlight fold change error bars the 95% CI.

The four miRNA that were confirmed to be differentially expressed by both the array and qRT-PCR experiments (hsa-miR-99b, hsa-miR-330-3p, hsa-miR-126, hsa-miR-30c) were interrogated in RNA extracted from these 4 major blood leukocyte populations. Three of the miRNA tested (hsa-miR-99b, hsa-miR-126 and hsa-miR-330-3p) demonstrated significant up-regulated expression in one or more PBMC subset in the CFS/ME population. Hsa-miR-30c exhibited no significant difference in expression between CFS/ME and control cell populations. The greatest difference in expression between population groups was in the expression of hsa-miR-99b in NK cells which demonstrated a 2 fold increase in CFS/ME samples. This miRNA was also up regulated 1.6 fold in B-cells, but remained unchanged in T-cells and monocytes. Hsa-miR-330-3p was also significantly up regulated in NK cells but to a much lower degree, exhibiting a 1.3 fold increase. Hsa-miR-126 was up regulated in monocytes by 1.45 fold in the CFS/ME group (Fig 4). These data identified that the CFS/ME-associated dysregulation of miRNA is leukocyte population specific, and suggest that dysfunction of blood B-cell, NK cells and monocytes contribute to disease pathology.

### Hsa-miR-99b and hsa-miR-330-3p regulate pathways contributing to NK cell activation and effector function

To identify the gene targets of hsa-miR-99b and hsa-miR-330-3p in NK cells, precursor miRNA were transfected into NK cells isolated by negative magnetic bead selection from buffy coat derived PBMCs. NK cell purity post enrichment was 89.6% (0.7 +/- SD) determined by flow cytometry (data not shown).

Transfection of primary NK cells with pre-miR-99b compared with transfected nonsense control identified 37 genes with differential expression passing the inclusion criteria (P≤0.05 fold change ≥1.5). Twenty one genes exhibited down regulation, with fold changes between 1.5 and 1.8. Sixteen genes demonstrated increased expression between 1.5 and 2.6. Interferon gamma (IFN-Y), which is a key mediator of NK cell activation, was the most highly induced gene (2.6 fold). Granzyme B (GZMB), which encodes a component of the cytotoxic granules that NK cells release upon recognition of target cells, was induced 1.6 fold. Genes involved in cellular activation, vesicle formation, motility and modulation of the actin cytoskeleton were also up regulated (KLRF1, STOM, S100A4, FEZ1, ARPC3 and TPM3P5). Cathepsin L (CTSL) was the most down regulated gene by hsa-miR-99b. This protein is required for cleavage and activation of perforin & granzyme B within lytic vesicles [32]. CD6 and IL-8 (interleukin-8) were also down regulated (1.6 and 1.5 fold respectively). Reduced expression of these genes is associated with NK cell maturation following activation [33, 34]. Reduced expression was also identified in genes involved in the autophagy, WNT and AKT signalling pathways (ULK1, ATG2A, PMEPA1, DKK3, PDCD4).

To determine whether the genes regulated by hsa-miR-99b grouped to a specific functional class, we conducted an overrepresentation analysis which identified a significant enrichment for genes involved in processes and pathways of NK cell function, including cell activation, immune system processes, and cellular response to stimulus (S3 Table).

Transfection of primary NK cells with pre-miR-330-3p compared to a nonsense control produced only two changes; to SOD2 and MMP9 expression, both of which were down regulated by 1.6 fold (S4 Table). SOD2 expression impairs normal T-cell differentiation and cellular activation [35, 36] and reduced expression is associated impaired neutrophil effector function [37]. MMP9 regulates NK cell cytotoxicity and lymphocyte motility [38, 39].

These results demonstrated that miRNAs expressed at increased levels in NK cells taken from CFS/ME patients promote gene expression changes consistent with cellular activation but reduced effector function.

## Discussion

Current clinical diagnosis of CFS/ME is made based on well-defined clinical criteria [3–5, 40], however diagnosis also includes a process of exclusion of other causes of fatigue which can result in a prolonged time to diagnosis. Thus there is a real clinical need to identify diagnostic biomarkers in CFS/ME. We set out to study the role of miRNA in patients with an established diagnosis of CFS/ME, in order to determine whether these regulatory molecules were contributing to disease pathogenesis and could be candidate diagnostic biomarkers.

We identified over-expression of 34 miRNA not previously described in CFS/ME [41, 42] in peripheral blood samples taken from CFS/ME patients by microarray. This analysis robustly groups miRNA with coordinated expression (hsa-miR-107 and hsa-miR-103) and is highly homogenous across samples. In common with other CFS/ME studies of gene expression the miRNA profile did not separate CFS/ME patients from controls [7]. The study of Brenu and colleagues (41) analysed plasma samples from CFS/ME patients and identified deregulation of 19 human miRNA. Of these 19, only 12 were represented in our array, of which 8 had a fold-difference of ≥1.5 in CFS/ME as compared with normals, but all had insignificant P values, and so were not investigated further. The absence of concordance between the two studies is unsurprising, as cellular and plasma miRNA profiles from the same individual demonstrate limited overlap [43]; this limited overlap has been proposed to result from the fact that miRNA are loaded into secreted vesicles with different efficiencies or the variable stability of different miRNA in the extracellular environment [44]. The CFS/ME population exhibit a wide range of clinical symptom scores supporting subgroups of disease which have been described elsewhere [25] and may contribute to the incomplete resolution of samples in this analysis. This observation also would explain the poor correlation between clinical symptom score and miRNA expression and would suggest that validation in an expanded patient cohort would be desirable.

Confirmation of the array findings by qRT-PCR supported the up regulation of hsa-miR-99b, hsa-miR-330-3p, hsa-miR-126 and hsa-miR-30c in CFS/ME PBMCs. The level of confirmation between the two methodologies is modest (R = -0.59) when compared to studies of mRNA expression in CFS/ME [7, 25]. This discrepancy has been suggested to be due in part to the differing methodologies employed [45], with fold changes below +/-2 fold [46–48] and low abundance of transcript linked to poorer concordance between methods. This highlights a consistent problem in miRNA studies as the cut-off commonly taken for miRNA gene expression experiments is 1.5 fold change which may lead to exclusion of positive data [49].

The miRNA with increased expression in CFS/ME could potentially act as disease biomarkers which could be utilised to improve and expedite diagnosis. This has been previously attempted in a number of studies identifying potential biomarkers in the serum [9], cytokine expression [31], Dipeptidyl peptidase-4 (CD26) [50] and lymphocyte gene expression [6, 51, 52]. To determine whether the four up regulated miRNA were suitable markers to resolve CFS/ME subjects from a matched control cohort, a ROC analysis was performed. This demonstrated that all four markers could resolve patients from controls (AUC = 0.71–0.78) with a comparable specificity to published studies. These miRNA may be specific to CFS/ME patients exhibiting expression consistent with controls in fibromyalgia [53, 54] and depression [55, 56] patients, two conditions with symptoms in common with CFS/ME. However, validation in larger blinded cohorts of patients would need to be undertaken before the diagnostic utility of the miRNA identified in this study could be fully explored.

Peripheral blood lymphocytes have been intensively examined in CFS/ME patients identifying alterations in cytokine secretion and changes in cellular populations and activity [13, 57]. The cellular pathways and processes regulated by miRNA are frequently cell specific [58, 59]. To attempt to identify the leucocyte population contributing to altered miRNA expression in CFS/ME, the four miRNA with increased expression were quantified in PBMC fractions from a new patient cohort. Hsa-miR-126 demonstrated increased expression in monocytes. Although to date a specific defect in monocyte activity has not been described in CFS/ME, hsa-miR-126 has been demonstrated to down-regulate CRK protein expression. This protein inhibits cell invasion and migration [60] and has been demonstrated to control proliferation in dendritic cells [61], suggesting increased monocyte activity mediated by miRNA may be contributing to the pathogenesis of CFS/ME.

Hsa-miR-330-3p and hsa-miR-99b demonstrate increased expression in B cell and NK cell populations. These miRNA have been associated with cancer, endometriosis, cell fate decisions and angiogenesis [62–70]. Hsa-miR-99b has been demonstrated to target components of NF-kB, mTOR and AKT signalling pathways [71]. mTOR signalling is crucial for normal proliferative, cytotoxic and cytokine effector functions in healthy NK cells [72] and normal BCR (B cell receptor) signalling and proliferation [73] in B-cells. Increased hsa-miR-99b expression regulating mTOR supports the reduction in effector function observed in NK cells in CFS/ME patients seen in the literature [11, 13] and supports the deregulation of genes involved in B-cell maturation and development identified in gene expression studies [74]. Hsa-miR-330-3p has been demonstrated to regulate cellular motility by down regulating SP1 in prostate cancer cells [75], and proliferation by reducing CDC42 levels in colorectal cancer [76].

Hsa-miR-30c does not exhibit altered expression in the cellular fractions analysed suggesting the over-expression observed in the original PBMC cohort and may be cohort-specific. This demonstrates the necessity for validation of findings in separate unrelated cohorts or patients.

To study the effects of the altered miRNA expression identified in CFS/ME on cellular mRNA, we elected to transfect the miRNA with the greatest magnitude of over expression (hsa-miR-99b and hsa-miR-330-3p) into healthy NK cells, which are most consistently identified as functionally impaired in CFS/ME patients [11, 13, 14]. Interferon Gamma (IFN-γ) demonstrated the greatest degree of altered expression in response to hsa-miR-99b introduction with a 2.6 fold increase in expression. This gene encodes a cytokine secreted by NK cells with a crucial role in anti-viral, anti-tumour and immune regulatory effects combined with promoting NK cell activity [77]. IFN-γ has been measured in a number of studies in CFS/ME with variable results. Increased mRNA in NK cells from CFS/ME patients has been demonstrated [13], supported by an increase in circulating IFN-γ secreting CD3- CD56+ NK cells described in CFS/ME patients [78]. However plasma concentrations of IFN-γ were unchanged when analysed in 40 female CFS/ME patients and matched controls [31].

One consistent finding in CFS/ME patients is the reduction in the cytotoxic activity of NK cells [11, 13, 14], frequently associated with an alteration in the level of components of cytotoxic granules notably granzyme and perforin [13, 14]. In this study Granzyme B (GZMB) was significantly up regulated by hsa-miR-99b suggesting an enhanced cytotoxic capacity. However CTSL (Cathepsin L) exhibited the greatest degree of down regulation by hsa-miR-99b. This enzyme is required to process lytic granule components, granzyme and perforin from precursor to active forms suggesting an explanation for the increased GZMB mRNA seen here and in the literature leading to reduced effector function. This mechanism suggests an accumulation of precursor proteins for GZMB and perforin in lytic vesicles with reduced cytotoxic function which is supported by recent data which identified reduced NK cell cytotoxicity in CFS/ME patients without an associated impairment in degranulation [78]. Over expression of hsa-miR-99b and hsa-miR-330-3p induced a gene expression pattern consistent with NK cell activation increasing IFN- γ expression and genes involved in cellular motility, vesicle formation and modulation of the actin cytoskeleton (KLRF1, STOM, S100A4, FEZ1, ARPC3 and TPM3P5), confirmed by over representation analysis. Activated NK cells in the peripheral blood of CFS/ME patients have been postulated to result from incomplete resolution and control of viral infection which is the most commonly cited trigger for onset of CFS/ME symptoms. This data also demonstrates a molecular basis for the reduction in NK cytotoxicity seen in CFS/ME patients mediated by miRNA, via direct down regulation of enzymes required for processing cytotoxic vesicle components and down regulation of genes within Autophagy, WNT and AKT pathways (ULK1, ATG2A, PMEPA1, DKK3, PDCD4) required for normal proliferation and maturation of activated NK cells. Additional validation of the protein targets regulated by the identified miRNA would be desirable to fully define the miRNA mediated NK cell dysfunction in CFS/ME.

The data presented identifies deregulation of miRNA expression in the peripheral blood cells of CFS/ME patients which was confirmed in two patient cohorts in PBMCs and cellular subsets and identifies four potential diagnostic biomarkers. Natural killer cells demonstrated the greatest changes in miRNA expression with up regulation of hsa-miR-99b and hsa-miR-330-3p. These findings are functionally consistent with current understanding of the pathogenesis of CFS/ME. The work presented here provides novel evidence for an altered NK cell activation pathway in CFS/ME involving hsa-miR-99b and suggests a mechanism for the reduced effector function seen in CFS/ME which has not been demonstrated previously.

## Materials and Methods

### Ethical Approval

All participants gave informed written consent for the withdrawal of 25ml of blood to be tested for gene expression analysis, clinical analysis by self-report questionnaire and storage of plasma/serum. This study was approved by Wandsworth Research Ethics Committee (Approval Number 05/Q0803/137).

### Subject recruitment

CFS/ME subjects were recruited from two specialist CFS/ME centres located in Poole hospital, Dorset (N = 15) and St Helier hospital (N = 20), London following diagnosis by a clinical specialist and the exclusion of other possible sources of fatigue. All CFS/ME subjects selected for the study were diagnosed using the Fukuda clinical criteria for diagnosing CFS/ME [3]. These subjects also fulfilled the Canadian criteria for diagnosis of CFS/ME [79]. Patients with psychiatric disease were excluded from the study using the Minnesota International Neuropsychiatric Interview (MINI); all CFS/ME subjects included in this study were therefore free of major psychiatric disease and drug or alcohol abuse. Average disease duration was 6.8 yrs.

Thirty healthy normal blood donors were recruited from the East Dorset National Blood Service (NBS) and 20 volunteers working within St George’s hospital that responded to invitations to take part in the study. Restrictions on donation to the NBS were used to maintain consistency within this comparison group which have been published elsewhere [7]. For both CFS/ME and control subject groups, individuals who smoked within the previous year, who abused alcohol or other drugs, were currently taking (or were within 3 months of taking) antibiotics, steroids, cytotoxic drugs or antidepressants were excluded from the study to remove any confounding factors that have been demonstrated to alter gene expression [80, 81]. Blood tests for haematological, biochemical, and liver function revealed no abnormalities in all patients.

### Clinical Data

Clinical data was collected from all participants in the form of five self-report questionnaires: Chalder Fatigue Scale, Pittsburgh Sleep Quality Index, McGill Pain Questionnaire, SPHERE and Medical Outcomes Survey—Short Form -36 (SF-36). These questionnaires have been assessed for suitability in recording CFS/ME symptoms by the CDC CFS/ME working group [82] and have provided evidence for defined groups of CFS/ME based on gene expression data [24]. All parameters assessed demonstrated a significant difference (P>0.05 Students T-test) from the healthy participants in both study populations with the exception of SF-36 segments for mental health and role emotional which were similar between CFS and control groups.

### miRNA Bioarray

PBMCs were isolated in vaccutainer CPT Cell Preparation tubes with Sodium Citrate (BD biosciences, NJ, USA) from 16ml of blood. RNA was extracted by Trizol and enriched for the small RNA fraction (<40nt) using the Ambion Flash-PAGE system, prior to labelling and hybridisation of mature RNA samples to Ambion Bioarray V1.0 which assays 385 miRNA per sample. 15 CFS samples recruited from the Dorset CFS clinic were age and sex matched +/- 5 years 2:1 with 30 controls recruited in the same geographical location. Seven CFS samples were run in duplicate, batching samples in groups of 14 with technical replicates run in separate batches. Technical replicates demonstrated a high concordance R = 0.97 (+/- 0.03 SD). Matched samples were run on the same array slide to minimise variation between slides. Arrays were scanned using a Genetic Microsystems GMS 418 scanner with a laser intensity of 95%, at range of gain settings to avoid saturation of array spots. Intensity values were generated using Imagene V5.5 (BioDiscovery, USA) using automatic segmentation. Un-background corrected median intensity values were corrected using Microarray valuation imp (MAVI) Pro 2.6.0 software (MWG biotech AG Ebersburg, Germany), which conducts a linear regression analysis on three gain scans of an array image to impute the values at the periphery of detection. Data was then normalised by median scaling setting the median of each array to 100 and log transformed (base10) adapted from [83]. A significant change in expression between groups was defined as a fold change of ≥1.5 between group means, taking P-values below 0.05 as significant after Mann-Whitney U test and Benjamini and Hochberg FDR correction [84]. Gene expression must also be present in 50% of the samples tested. Matched samples P100_1 and C261 failed hybridisation and were excluded (GSE70371).

### Quantitative Polymerase Chain reaction (qRT-PCR)

miRNA expression changes observed by microarray were validated by quantitative reverse transcription qRT—PCR. cDNA was generated from 10 ng Total RNA with miRNA specific primers using the Taqman reverse transcription kit (Applied biosystems). cDNA (1.33 μL) was added to triplicate 20 μl PCR reactions with corresponding no-template and no-RT controls for each reaction. Hsa-miR-16 was used as the endogenous control in PBMC samples, CD3+ T-Cell, CD14+ Monocyte fractions. Hsa-let-7a was used for the endogenous control in CD19+ B-cell and CD56+ NK cell experiments after validation of 8 putative endogenous controls (hsa-miR-16, hsa-Let-7a, hsa-miR-106a, RNU6B, hsa-miR-154, hsa-miR-30e-3p and rno-miR-336) for stability of expression using gNorm [85] and normfinder algorithms [86]. Reactions were performed on the 7500 Fast real-time PCR system (Applied Biosystems) using the standard thermal cycler protocol. Average cT was generated from triplicate reactions with SD>0.5. The change in expression of each target gene was calculated relative to the endogenous control gene using the Relative quantification (RQ) method where RQ = 2^−ΔΔCT^. Fold change ratio were calculated by diving groups means.

### Statistical analysis

Statistical analysis of miRNA data was performed in Genespring V 7.3.1 (Agilent technologies, USA) & Bead studio and Genespring V 10 for the mRNA array data. Pearson correlations and ROC analysis were performed in Prism V5.03 and Excel. Clustering of gene expression values was carried out in cluster V3.0, prior to visualising in TreeView V1.60. Similarity between miRNA’s and samples was determined using Euclidean distance; Average linkage was used to calculate the distance between samples or miRNA.

### Gene Ontology

miRNA predicted targets and gene expression data were subjected to an over-representation analysis using Panther gene classification software (www.pantherdb.org) to identify enrichment for Pathways, Molecular function and Biological processes. Statistical significance was assigned by binomial test comparing the number of genes highlighted in a particular pathway, to a list of genes of the same size randomly selected from the genome and classified in the same manner. Data with P-values > 0.01 were excluded.

### Enrichment for cellular populations

PBMCs were isolated from blood samples and buffy coats by density gradient centrifugation. Monocytes (CD14+), B-cells (CD19+), NK cells (CD56+) and T-cells (CD3+) were isolated using Miltenyi Macs beads and columns sequentially labelling and positively selecting populations. RNA was isolated using trizol determining quality and quantity by nanodrop and Agilent bioanalyser. NK cells for the functional target characterisation were isolated from healthy buffy coats purchased from the NBS using the negative selection NK cell isolation kit II (Miltenyi biotec).

### Flow Cytometry

Cellular purity post enrichment was determined for each fraction by flow cytometry. For each population 2.5x10^5^ cells were labelled with 5μl of corresponding PE conjugated antibody incubating at 4°C for 30 minutes. The following antibodies were used: CD19-PE (LT19), CD56-PE (REA196), CD14-PE (TÜK4), CD3-PE (BW264/56), IgG1-PE and IgG2-P (miltenyi biotec).

### Over expression of miRNA precursors in healthy NK cells

NK cells were transfected by electroporation using the Amaxa Human NK cell nucleofector kit (Lonza) with 100nM of hsa-miR-99b, hsa-miR-330-3P or non-sense control precursors (Ambion). These conditions generated the greatest reduction in PTK9 mRNA of 71% after hsa-miR-1 transfection at 24 hours incubation (Pre-miR miRNA starter kit, Ambion) when assessed by qPCR (data not shown). 2x10^6^ transfected NK cells were incubated at 37°C with 5% CO^2^ in RPMI 1640 cell culture media supplemented with 10% Fetal bovine serum (Invitrogen), 100μg/ml Streptomycin, 100U/ml penicillin, 2mM GlutaMAX (Invitrogen) and 200 U/ml IL-2 (Miltenyi). Triplicate transfections were prepared for each condition. 24 hours post transfection cells were washed twice with PBS and RNA isolated with Trizol.

### mRNA Analysis by Illumina HT12 V3 bead array

The Illumina TotalPrep RNA Amplification kit (Ambion PNIL1791M) was used to generate fragmented biotin labelled cRNA for hybridisation to Illumina HT12 V3 bead arrays. 750ng RNA was prepared for each of the three transfection conditions. Bead chips were scanned on the iScan scanner (Illumina San Diego, CA). Prior to the detection of differentially expressed genes the data was locally background corrected for each bead, averages were generated for beads with the same probe and outliers removed. This is an automatic correction performed by the iScan software (Illumina, San Diego, CA). The data was quality assessed in Genome studio v1.1.1 (Illumina, San Diego, CA). The bead array data was imported into Genespring V10 for normalisation and detection of differentially expressed genes. Quantile normalisation was used combined with an additional normalisation to the non-sense transfected samples which represents the baseline expression for the experiment. The data was filtered to remove probes where detection of an expressed signal was incomplete in the biological replicates. The difference in expression between target gene transfection and non-sense control was calculated by subtracting the log transformed group means for each group to generate a fold change ratio. Genes were selected for further analysis if they demonstrated a P-value after T-testing of ≤0.05 and passed the fold change cut-off of a change in expression over 1.5 fold (GSE69555).

## Supporting Information

S1 Table Patient and control characteristics summarizing clinical questionnaire data group sizes, means, standard deviation and t-test P-value for each questionnaire. (DOC)

S2 Table 34 miRNA with differential expression between CFS/ME and matched controls; highlighting group size and means, fold change and P-values from Mann-Whitney U test before and after FDR correction. (DOC)

S3 Table Panther classification analysis of 39 genes deregulated by hsa-miR-99b and hsa-miR-330-3p in primary NK cells highlighting classification by biological process. (DOC)

S4 Table Genes demonstrating differential expression in primary NK cells after transfection with either pre-miR-99b or pre-miR-330-3P relative to a non-sense control transfection. Genes demonstrating fold changes >1.5 and P≤0.05 were selected for further analysis.
(DOC)

## References

[1] group Tcmosw. A report of the CFS/ME Working Group. 2002.

[2] Papanicolaou DA, Amsterdam JD, Levine S, McCann SM, Moore RC, Newbrand CH, et al Neuroendocrine aspects of chronic fatigue syndrome. Neuroimmunomodulation. 2004;11(2):65–74. Epub 2004/02/06. 10.1159/000075315 [pii]. . 14758052

[3] Fukuda K, Straus SE, Hickie I, Sharpe MC, Dobbins JG, Komaroff A. The chronic fatigue syndrome: a comprehensive approach to its definition and study. International Chronic Fatigue Syndrome Study Group. Ann Intern Med. 1994;121(12):953–9. Epub 1994/12/15. . 7978722 10.7326/0003-4819-121-12-199412150-00009

[4] Sharpe MC, Archard LC, Banatvala JE, Borysiewicz LK, Clare AW, David A, et al A report—chronic fatigue syndrome: guidelines for research. J R Soc Med. 1991;84(2):118–21. Epub 1991/02/01. 1999813 10.1177/014107689108400224 PMC1293107

[5] Carruthers BM, Kumar Jain A., De Meirleir K.L., Peterson D.L., Klimas N.G., lerner M., Bested A.C., Flor-Henry P., Joshi P., Powels P., Sherkey J.A., van de Sande M.I. Myalgic Encephalomyelitis/ Chronic Fatigue Syndrome: Clinical Working Case Definition, Diagnostic and Treatment Protocols. Journal of Chronic Fatigue Syndrome. 2003;11(1):7.

[6] Frampton D, Kerr J, Harrison TJ, Kellam P. Assessment of a 44 gene classifier for the evaluation of chronic fatigue syndrome from peripheral blood mononuclear cell gene expression. PLoS One. 2011;6(3):e16872 Epub 2011/04/12. 10.1371/journal.pone.0016872 21479222 PMC3068152

[7] Kaushik N, Fear D, Richards SC, McDermott CR, Nuwaysir EF, Kellam P, et al Gene expression in peripheral blood mononuclear cells from patients with chronic fatigue syndrome. J Clin Pathol. 2005;58(8):826–32. Epub 2005/07/29. 58/8/826 [pii] 10.1136/jcp.2005.025718 16049284 PMC1770875

[8] Vernon SD, Unger ER, Dimulescu IM, Rajeevan M, Reeves WC. Utility of the blood for gene expression profiling and biomarker discovery in chronic fatigue syndrome. Dis Markers. 2002;18(4):193–9. Epub 2003/02/19. 12590173 10.1155/2002/892374 PMC3851413

[9] Fletcher MA, Rosenthal M, Antoni M, Ironson G, Zeng XR, Barnes Z, et al Plasma neuropeptide Y: a biomarker for symptom severity in chronic fatigue syndrome. Behav Brain Funct. 2010;6:76. 21190576 10.1186/1744-9081-6-76 PMC3024290

[10] Holgate ST, Komaroff AL, Mangan D, Wessely S. Chronic fatigue syndrome: understanding a complex illness. Nat Rev Neurosci. 2011;12(9):539–44. Epub 2011/07/28. 10.1038/nrn3087 [pii]. . 21792218

[11] Klimas NG, Salvato FR, Morgan R, Fletcher MA. Immunologic abnormalities in chronic fatigue syndrome. J Clin Microbiol. 1990;28(6):1403–10. Epub 1990/06/01. 2166084 10.1128/jcm.28.6.1403-1410.1990 PMC267940

[12] Brenu EW, Huth TK, Hardcastle SL, Fuller K, Kaur M, Johnston S, et al Role of adaptive and innate immune cells in chronic fatigue syndrome/myalgic encephalomyelitis. Int Immunol. 2014 Epub 2013/12/18. dxt068 [pii] 10.1093/intimm/dxt068 . 24343819

[13] Brenu EW, van Driel ML, Staines DR, Ashton KJ, Ramos SB, Keane J, et al Immunological abnormalities as potential biomarkers in Chronic Fatigue Syndrome/Myalgic Encephalomyelitis. J Transl Med. 2011;9:81 Epub 2011/05/31. 10.1186/1479-5876-9-81 [pii]. 21619669 PMC3120691

[14] Maher KJ, Klimas NG, Fletcher MA. Chronic fatigue syndrome is associated with diminished intracellular perforin. Clin Exp Immunol. 2005;142(3):505–11. Epub 2005/11/22. CEI2935 [pii] 10.1111/j.1365-2249.2005.02935.x 16297163 PMC1440524

[15] Whistler T, Unger ER, Nisenbaum R, Vernon SD. Integration of gene expression, clinical, and epidemiologic data to characterize Chronic Fatigue Syndrome. J Transl Med. 2003;1(1):10. 14641939 10.1186/1479-5876-1-10 PMC305360

[16] Whistler T, Taylor R, Craddock RC, Broderick G, Klimas N, Unger ER. Gene expression correlates of unexplained fatigue. Pharmacogenomics. 2006;7(3):395–405. Epub 2006/04/14. 10.2217/14622416.7.3.395 . 16610950

[17] Whistler T, Jones JF, Unger ER, Vernon SD. Exercise responsive genes measured in peripheral blood of women with chronic fatigue syndrome and matched control subjects. BMC Physiol. 2005;5(1):5 Epub 2005/03/26. 1472-6793-5-5 [pii] 10.1186/1472-6793-5-5 15790422 PMC1079885

[18] Powell R, Ren J, Lewith G, Barclay W, Holgate S, Almond J. Identification of novel expressed sequences, up-regulated in the leucocytes of chronic fatigue syndrome patients. Clin Exp Allergy. 2003;33(10):1450–6. Epub 2003/10/02. 1745 [pii]. . 14519154 10.1046/j.1365-2222.2003.01745.x

[19] Broderick G, Craddock RC, Whistler T, Taylor R, Klimas N, Unger ER. Identifying illness parameters in fatiguing syndromes using classical projection methods. Pharmacogenomics. 2006;7(3):407–19. Epub 2006/04/14. 10.2217/14622416.7.3.407 . 16610951

[20] Carmel L, Efroni S, White PD, Aslakson E, Vollmer-Conna U, Rajeevan MS. Gene expression profile of empirically delineated classes of unexplained chronic fatigue. Pharmacogenomics. 2006;7(3):375–86. Epub 2006/04/14. 10.2217/14622416.7.3.375 . 16610948

[21] Fang H, Xie Q, Boneva R, Fostel J, Perkins R, Tong W. Gene expression profile exploration of a large dataset on chronic fatigue syndrome. Pharmacogenomics. 2006;7(3):429–40. Epub 2006/04/14. 10.2217/14622416.7.3.429 . 16610953

[22] Fostel J, Boneva R, Lloyd A. Exploration of the gene expression correlates of chronic unexplained fatigue using factor analysis. Pharmacogenomics. 2006;7(3):441–54. Epub 2006/04/14. 10.2217/14622416.7.3.441 . 16610954

[23] Grans H, Nilsson P, Evengard B. Gene expression profiling in the chronic fatigue syndrome. J Intern Med. 2005;258(4):388–90. Epub 2005/09/17. JIM1548 [pii] 10.1111/j.1365-2796.2005.01548.x . 16164580

[24] Kerr JR, Petty R, Burke B, Gough J, Fear D, Sinclair LI, et al Gene expression subtypes in patients with chronic fatigue syndrome/myalgic encephalomyelitis. J Infect Dis. 2008;197(8):1171–84. Epub 2008/05/09. 10.1086/533453 . 18462164

[25] Kerr JR, Burke B, Petty R, Gough J, Fear D, Mattey DL, et al Seven genomic subtypes of chronic fatigue syndrome/myalgic encephalomyelitis: a detailed analysis of gene networks and clinical phenotypes. J Clin Pathol. 2008;61(6):730–9. Epub 2007/12/07. jcp.2007.053553 [pii] 10.1136/jcp.2007.053553 . 18057078

[26] Kerr JR. Gene profiling of patients with chronic fatigue syndrome/myalgic encephalomyelitis. Curr Rheumatol Rep. 2008;10(6):482–91. Epub 2008/11/15. 19007540 10.1007/s11926-008-0079-5

[27] Li QJ, Chau J, Ebert PJ, Sylvester G, Min H, Liu G, et al miR-181a is an intrinsic modulator of T cell sensitivity and selection. Cell. 2007;129(1):147–61. Epub 2007/03/27. S0092-8674(07)00319-4 [pii] 10.1016/j.cell.2007.03.008 . 17382377

[28] Chen CZ, Li L, Lodish HF, Bartel DP. MicroRNAs modulate hematopoietic lineage differentiation. Science. 2004;303(5654):83–6. Epub 2003/12/06. 10.1126/science.1091903 [pii]. . 14657504

[29] Roush S, Slack FJ. The let-7 family of microRNAs. Trends Cell Biol. 2008;18(10):505–16. Epub 2008/09/09. 10.1016/j.tcb.2008.07.007 S0962-8924(08)00210-9 [pii]. . 18774294

[30] Martello G, Rosato A, Ferrari F, Manfrin A, Cordenonsi M, Dupont S, et al A MicroRNA targeting dicer for metastasis control. Cell. 2010;141(7):1195–207. Epub 2010/07/07. 10.1016/j.cell.2010.05.017 S0092-8674(10)00553-2 [pii]. . 20603000

[31] Fletcher MA, Zeng XR, Barnes Z, Levis S, Klimas NG. Plasma cytokines in women with chronic fatigue syndrome. J Transl Med. 2009;7:96 Epub 2009/11/17. 10.1186/1479-5876-7-96 [pii]. 19909538 PMC2779802

[32] Meade JL, de Wynter EA, Brett P, Sharif SM, Woods CG, Markham AF, et al A family with Papillon-Lefevre syndrome reveals a requirement for cathepsin C in granzyme B activation and NK cell cytolytic activity. Blood. 2006;107(9):3665–8. Epub 2006/01/18. 2005-03-1140 [pii] 10.1182/blood-2005-03-1140 . 16410452

[33] Braun M, Muller B, ter Meer D, Raffegerst S, Simm B, Wilde S, et al The CD6 scavenger receptor is differentially expressed on a CD56 natural killer cell subpopulation and contributes to natural killer-derived cytokine and chemokine secretion. J Innate Immun. 2011;3(4):420–34. Epub 2010/12/24. 10.1159/000322720 [pii]. . 21178331

[34] Montaldo E, Vitale C, Cottalasso F, Conte R, Glatzer T, Ambrosini P, et al Human NK cells at early stages of differentiation produce CXCL8 and express CD161 molecule that functions as an activating receptor. Blood. 2012;119(17):3987–96. Epub 2012/03/10. 10.1182/blood-2011-09-379693 [pii]. . 22403260

[35] Kaminski MM, Roth D, Sass S, Sauer SW, Krammer PH, Gulow K. Manganese superoxide dismutase: a regulator of T cell activation-induced oxidative signaling and cell death. Biochim Biophys Acta. 2012;1823(5):1041–52. Epub 2012/03/21. 10.1016/j.bbamcr.2012.03.003 S0167-4889(12)00063-8 [pii]. . 22429591

[36] Case AJ, McGill JL, Tygrett LT, Shirasawa T, Spitz DR, Waldschmidt TJ, et al Elevated mitochondrial superoxide disrupts normal T cell development, impairing adaptive immune responses to an influenza challenge. Free Radic Biol Med. 2011;50(3):448–58. Epub 2010/12/07. 10.1016/j.freeradbiomed.2010.11.025 S0891-5849(10)01397-3 [pii]. 21130157 PMC3026081

[37] Olsson J, Jacobson TA, Paulsson JM, Dadfar E, Moshfegh A, Jacobson SH, et al Expression of neutrophil SOD2 is reduced after lipopolysaccharide stimulation: a potential cause of neutrophil dysfunction in chronic kidney disease. Nephrol Dial Transplant. 2011;26(7):2195–201. Epub 2010/11/04. 10.1093/ndt/gfq673 [pii]. . 21045076

[38] Liu Q, Sun Y, Rihn S, Nolting A, Tsoukas PN, Jost S, et al Matrix metalloprotease inhibitors restore impaired NK cell-mediated antibody-dependent cellular cytotoxicity in human immunodeficiency virus type 1 infection. J Virol. 2009;83(17):8705–12. Epub 2009/06/26. 10.1128/JVI.02666-08 JVI.02666-08[pii]. 19553339 PMC2738177

[39] Wu B, Crampton SP, Hughes CC. Wnt signaling induces matrix metalloproteinase expression and regulates T cell transmigration. Immunity. 2007;26(2):227–39. Epub 2007/02/20. S1074-7613(07)00137-9 [pii] 10.1016/j.immuni.2006.12.007 17306568 PMC1855210

[40] Carruthers BM. Definitions and aetiology of myalgic encephalomyelitis: how the Canadian consensus clinical definition of myalgic encephalomyelitis works. J Clin Pathol. 2007;60(2):117–9. Epub 2006/08/29. jcp.2006.042754 [pii] 10.1136/jcp.2006.042754 16935963 PMC1860613

[41] Brenu EW, Ashton KJ, Batovska J, Staines DR, Marshall-Gradisnik SM. High-throughput sequencing of plasma microRNA in chronic fatigue syndrome/myalgic encephalomyelitis. PLoS One. 2014;9(9):e102783 Epub 2014/09/23. 10.1371/journal.pone.0102783 PONE-D-13-54226 [pii]. 25238588 PMC4169517

[42] Brenu EW, Ashton KJ, van Driel M, Staines DR, Peterson D, Atkinson GM, et al Cytotoxic lymphocyte microRNAs as prospective biomarkers for Chronic Fatigue Syndrome/Myalgic Encephalomyelitis. J Affect Disord. 2012;141(2–3):261–9. Epub 2012/05/11. 10.1016/j.jad.2012.03.037 S0165-0327(12)00237-6 [pii]. . 22572093

[43] Turchinovich A, Burwinkel B. Distinct AGO1 and AGO2 associated miRNA profiles in human cells and blood plasma. RNA Biol. 2012;9(8):1066–75. 10.4161/rna.21083 22858679 PMC3551861

[44] Valadi H, Ekstrom K, Bossios A, Sjostrand M, Lee JJ, Lotvall JO. Exosome-mediated transfer of mRNAs and microRNAs is a novel mechanism of genetic exchange between cells. Nat Cell Biol. 2007;9(6):654–9. 10.1038/ncb1596 . 17486113

[45] Sato F, Tsuchiya S, Terasawa K, Tsujimoto G. Intra-platform repeatability and inter-platform comparability of microRNA microarray technology. PLoS One. 2009;4(5):e5540 Epub 2009/05/14. 10.1371/journal.pone.0005540 19436744 PMC2677665

[46] Wurmbach E, Yuen T, Sealfon SC. Focused microarray analysis. Methods. 2003;31(4):306–16. Epub 2003/11/05. S1046202303001610 [pii]. . 14597315 10.1016/s1046-2023(03)00161-0

[47] Etienne W, Meyer MH, Peppers J, Meyer RA Jr. Comparison of mRNA gene expression by RT-PCR and DNA microarray. Biotechniques. 2004;36(4):618–20, 22, 24–6. Epub 2004/04/20. . 15088380 10.2144/04364ST02

[48] Rajeevan MS, Vernon SD, Taysavang N, Unger ER. Validation of array-based gene expression profiles by real-time (kinetic) RT-PCR. J Mol Diagn. 2001;3(1):26–31. Epub 2001/02/28. S1525-1578(10)60646-0 [pii] 10.1016/S1525-1578(10)60646-0 11227069 PMC1907344

[49] Mestdagh P, Van Vlierberghe P, De Weer A, Muth D, Westermann F, Speleman F, et al A novel and universal method for microRNA RT-qPCR data normalization. Genome Biol. 2009;10(6):R64 Epub 2009/06/18. 10.1186/gb-2009-10-6-r64 [pii]. 19531210 PMC2718498

[50] Fletcher MA, Zeng XR, Maher K, Levis S, Hurwitz B, Antoni M, et al Biomarkers in chronic fatigue syndrome: evaluation of natural killer cell function and dipeptidyl peptidase IV/CD26. PLoS One. 2010;5(5):e10817 Epub 2010/06/04. 10.1371/journal.pone.0010817 20520837 PMC2876037

[51] Steinau M, Unger ER, Vernon SD, Jones JF, Rajeevan MS. Differential-display PCR of peripheral blood for biomarker discovery in chronic fatigue syndrome. J Mol Med (Berl). 2004;82(11):750–5. Epub 2004/10/19. 10.1007/s00109-004-0586-4 . 15490094

[52] Light AR, White AT, Hughen RW, Light KC. Moderate exercise increases expression for sensory, adrenergic, and immune genes in chronic fatigue syndrome patients but not in normal subjects. J Pain. 2009;10(10):1099–112. Epub 2009/08/04. 10.1016/j.jpain.2009.06.003 S1526-5900(09)00574-4 [pii]. 19647494 PMC2757484

[53] Bjersing JL, Lundborg C, Bokarewa MI, Mannerkorpi K. Profile of cerebrospinal microRNAs in fibromyalgia. PLoS One. 2013;8(10):e78762 Epub 2013/11/10. 10.1371/journal.pone.0078762 PONE-D-13-23414 [pii]. 24205312 PMC3808359

[54] Cerda-Olmedo G, Mena-Duran AV, Monsalve V, Oltra E. Identification of a MicroRNA Signature for the Diagnosis of Fibromyalgia. PLoS One. 2015;10(3):e0121903. 25803872 10.1371/journal.pone.0121903 PMC4372601

[55] Wan Y, Liu Y, Wang X, Wu J, Liu K, Zhou J, et al Identification of Differential MicroRNAs in Cerebrospinal Fluid and Serum of Patients with Major Depressive Disorder. PLoS One. 2015;10(3):e0121975. 25763923 10.1371/journal.pone.0121975 PMC4357380

[56] Fan HM, Sun XY, Guo W, Zhong AF, Niu W, Zhao L, et al Differential expression of microRNA in peripheral blood mononuclear cells as specific biomarker for major depressive disorder patients. J Psychiatr Res. 2014;59:45–52. Epub 2014/09/10. 10.1016/j.jpsychires.2014.08.007 S0022-3956(14)00243-X [pii]. . 25201637

[57] Brenu EW, van Driel ML, Staines DR, Ashton KJ, Hardcastle SL, Keane J, et al Longitudinal investigation of natural killer cells and cytokines in chronic fatigue syndrome/myalgic encephalomyelitis. J Transl Med. 2012;10:88 Epub 2012/05/11. 10.1186/1479-5876-10-88 [pii]. 22571715 PMC3464733

[58] Ramkissoon SH, Mainwaring LA, Ogasawara Y, Keyvanfar K, McCoy JP Jr., Sloand EM, et al Hematopoietic-specific microRNA expression in human cells. Leuk Res. 2006;30(5):643–7. Epub 2005/10/18. S0145-2126(05)00356-5 [pii] 10.1016/j.leukres.2005.09.001 . 16226311

[59] Jopling CL, Yi M, Lancaster AM, Lemon SM, Sarnow P. Modulation of hepatitis C virus RNA abundance by a liver-specific MicroRNA. Science. 2005;309(5740):1577–81. Epub 2005/09/06. 309/5740/1577 [pii] 10.1126/science.1113329 . 16141076

[60] Feng R, Chen X, Yu Y, Su L, Yu B, Li J, et al miR-126 functions as a tumour suppressor in human gastric cancer. Cancer Lett. 2010;298(1):50–63. Epub 2010/07/14. 10.1016/j.canlet.2010.06.004 S0304-3835(10)00316-2 [pii]. . 20619534

[61] Agudo J, Ruzo A, Tung N, Salmon H, Leboeuf M, Hashimoto D, et al The miR-126-VEGFR2 axis controls the innate response to pathogen-associated nucleic acids. Nat Immunol. 2014;15(1):54–62. Epub 2013/11/26. 10.1038/ni.2767 [pii]. 24270517 PMC3896265

[62] Jeyapalan Z, Deng Z, Shatseva T, Fang L, He C, Yang BB. Expression of CD44 3'-untranslated region regulates endogenous microRNA functions in tumorigenesis and angiogenesis. Nucleic Acids Res. 2011;39(8):3026–41. Epub 2010/12/15. 10.1093/nar/gkq1003 [pii]. 21149267 PMC3082902

[63] Lee KH, Chen YL, Yeh SD, Hsiao M, Lin JT, Goan YG, et al MicroRNA-330 acts as tumor suppressor and induces apoptosis of prostate cancer cells through E2F1-mediated suppression of Akt phosphorylation. Oncogene. 2009;28(38):3360–70. Epub 2009/07/15. 10.1038/onc.2009.192 [pii]. . 19597470

[64] Pang Y, Young CY, Yuan H. MicroRNAs and prostate cancer. Acta Biochim Biophys Sin (Shanghai). 2010;42(6):363–9. Epub 2010/06/12. . 20539944 10.1093/abbs/gmq038

[65] Dalmasso G, Nguyen HT, Yan Y, Laroui H, Srinivasan S, Sitaraman SV, et al MicroRNAs determine human intestinal epithelial cell fate. Differentiation. 2010;80(2–3):147–54. Epub 2010/07/20. 10.1016/j.diff.2010.06.005 S0301-4681(10)00068-X [pii]. 20638171 PMC2943016

[66] Gougelet A, Pissaloux D, Besse A, Perez J, Duc A, Dutour A, et al Micro-RNA profiles in osteosarcoma as a predictive tool for ifosfamide response. Int J Cancer. 2011;129(3):680–90. Epub 2010/10/16. 10.1002/ijc.25715 . 20949564

[67] Hisaoka M, Matsuyama A, Nagao Y, Luan L, Kuroda T, Akiyama H, et al Identification of altered MicroRNA expression patterns in synovial sarcoma. Genes Chromosomes Cancer. 2011;50(3):137–45. Epub 2011/01/08. 10.1002/gcc.20837 . 21213367

[68] Lionetti M, Biasiolo M, Agnelli L, Todoerti K, Mosca L, Fabris S, et al Identification of microRNA expression patterns and definition of a microRNA/mRNA regulatory network in distinct molecular groups of multiple myeloma. Blood. 2009;114(25):e20–6. Epub 2009/10/23. 10.1182/blood-2009-08-237495 [pii]. . 19846888

[69] Ohlsson Teague EM, Van der Hoek KH, Van der Hoek MB, Perry N, Wagaarachchi P, Robertson SA, et al MicroRNA-regulated pathways associated with endometriosis. Mol Endocrinol. 2009;23(2):265–75. Epub 2008/12/17. 10.1210/me.2008-0387 [pii]. . 19074548 PMC5419313

[70] Wu W, Lin Z, Zhuang Z, Liang X. Expression profile of mammalian microRNAs in endometrioid adenocarcinoma. Eur J Cancer Prev. 2009;18(1):50–5. Epub 2008/12/17. 10.1097/CEJ.0b013e328305a07a 00008469-200902000-00008 [pii]. . 19077565

[71] Jin Y, Tymen SD, Chen D, Fang ZJ, Zhao Y, Dragas D, et al MicroRNA-99 family targets AKT/mTOR signaling pathway in dermal wound healing. PLoS One. 2013;8(5):e64434 Epub 2013/06/01. 10.1371/journal.pone.0064434 PONE-D-12-39965 [pii]. 23724047 PMC3665798

[72] Nandagopal N, Ali AK, Komal AK, Lee SH. The Critical Role of IL-15-PI3K-mTOR Pathway in Natural Killer Cell Effector Functions. Front Immunol. 2014;5:187 Epub 2014/05/06. 10.3389/fimmu.2014.00187 24795729 PMC4005952

[73] Donahue AC, Fruman DA. Distinct signaling mechanisms activate the target of rapamycin in response to different B-cell stimuli. Eur J Immunol. 2007;37(10):2923–36. Epub 2007/08/29. 10.1002/eji.200737281 . 17724683

[74] Aspler AL, Bolshin C, Vernon SD, Broderick G. Evidence of inflammatory immune signaling in chronic fatigue syndrome: A pilot study of gene expression in peripheral blood. Behav Brain Funct. 2008;4:44 Epub 2008/09/30. 10.1186/1744-9081-4-44 [pii]. 18822143 PMC2569951

[75] Mao Y, Chen H, Lin Y, Xu X, Hu Z, Zhu Y, et al microRNA-330 inhibits cell motility by downregulating Sp1 in prostate cancer cells. Oncol Rep. 2013;30(1):327–33. Epub 2013/05/15. 10.3892/or.2013.2452 . 23670210

[76] Li Y, Zhu X, Xu W, Wang D, Yan J. miR-330 regulates the proliferation of colorectal cancer cells by targeting Cdc42. Biochem Biophys Res Commun. 2013;431(3):560–5. Epub 2013/01/23. 10.1016/j.bbrc.2013.01.016 S0006-291X(13)00052-1 [pii]. . 23337504

[77] Schroder K, Hertzog PJ, Ravasi T, Hume DA. Interferon-gamma: an overview of signals, mechanisms and functions. J Leukoc Biol. 2004;75(2):163–89. Epub 2003/10/04. 10.1189/jlb.0603252 [pii]. . 14525967

[78] Brenu EW, Huth TK, Hardcastle SL, Fuller K, Kaur M, Johnston S, et al Role of adaptive and innate immune cells in chronic fatigue syndrome/myalgic encephalomyelitis. Int Immunol. 2014;26(4):233–42. Epub 2013/12/18. 10.1093/intimm/dxt068 [pii]. . 24343819

[79] Carruthers BM. Definitions and aetiology of myalgic encephalomyelitis: how the Canadian consensus clinical definition of myalgic encephalomyelitis works. Journal of Clinical Pathology. 2007;60(2):117–9. 16935963 10.1136/jcp.2006.042754 PMC1860613

[80] Melas PA, Rogdaki M, Lennartsson A, Bjork K, Qi H, Witasp A, et al Antidepressant treatment is associated with epigenetic alterations in the promoter of P11 in a genetic model of depression. Int J Neuropsychopharmacol. 2012;15(5):669–79. Epub 2011/06/21. 10.1017/S1461145711000940 [pii]. . 21682946

[81] Laytragoon-Lewin N, Bahram F, Rutqvist LE, Turesson I, Lewin F. Direct effects of pure nicotine, cigarette smoke extract, Swedish-type smokeless tobacco (Snus) extract and ethanol on human normal endothelial cells and fibroblasts. Anticancer Res. 2011;31(5):1527–34. Epub 2011/05/28. 31/5/1527 [pii]. . 21617206

[82] Reeves WC, Lloyd A, Vernon SD, Klimas N, Jason LA, Bleijenberg G, et al Identification of ambiguities in the 1994 chronic fatigue syndrome research case definition and recommendations for resolution. BMC Health Serv Res. 2003;3(1):25 Epub 2004/01/02. 10.1186/1472-6963-3-25 [pii]. 14702202 PMC317472

[83] Shingara J, Keiger K, Shelton J, Laosinchai-Wolf W, Powers P, Conrad R, et al An optimized isolation and labeling platform for accurate microRNA expression profiling. RNA. 2005;11(9):1461–70. Epub 2005/07/27. rna.2610405 [pii] 10.1261/rna.2610405 16043497 PMC1370829

[84] Keller A, Leidinger P, Lange J, Borries A, Schroers H, Scheffler M, et al Multiple sclerosis: microRNA expression profiles accurately differentiate patients with relapsing-remitting disease from healthy controls. PLoS One. 2009;4(10):e7440 Epub 2009/10/14. 10.1371/journal.pone.0007440 19823682 PMC2757919

[85] Vandesompele J, De Preter K, Pattyn F, Poppe B, Van Roy N, De Paepe A, et al Accurate normalization of real-time quantitative RT-PCR data by geometric averaging of multiple internal control genes. Genome Biol. 2002;3(7):RESEARCH0034 Epub 2002/08/20. 12184808 10.1186/gb-2002-3-7-research0034 PMC126239

[86] Andersen CL, Jensen JL, Orntoft TF. Normalization of real-time quantitative reverse transcription-PCR data: a model-based variance estimation approach to identify genes suited for normalization, applied to bladder and colon cancer data sets. Cancer Res. 2004;64(15):5245–50. Epub 2004/08/04. 10.1158/0008-5472.CAN-04-0496 64/15/5245[pii]. . 15289330

