# Supplementary material for: MicroRNAs hsa-miR-99b, hsa-miR-330, hsa-miR-126 and hsa-miR-30c: Potential Diagnostic Biomarkers in Natural Killer (NK) Cells of Patients with Chronic Fatigue Syndrome (CFS)/ Myalgic Encephalomyelitis (ME)
Source: PLoS One. 2016 Mar 11;11(3):e0150904. doi: 10.1371/journal.pone.0150904 (PMC4788442; doi:10.1371/journal.pone.0150904)
Supplement: S1 Table — (DOC) [file pone.0150904.s001.doc]

**Table S1.** Patient and control characteristics summarizing clinical questionnaire data group sizes, means, standard deviation and T-test P-value for each questionnaire total.

|  |  | **Participants** | **Female** | **Male** | **Average age (Years)** | **Questionnaire responders** | **Chalder Fatigue** | **Pittsburgh sleep Index score** | **SPHERE** | **McGill Pain** | **SF-36** |
| --- | --- | --- | --- | --- | --- | --- | --- | --- | --- | --- | --- |
| **Microarray Cohort** | **CFS** | 15 | 12 | 3 | 43.8 | 15 |  |  |  |  |  |
|  | **Control** | 30 | 22 | 6 | 45.3 | 28 |  |  |  |  |  |
| **Fractionated lymphocyte cohort** | **CFS** | 20 | 16 | 4 | 37.55 | 15 |  |  |  |  |  |
|  | **Control** | 20 | 16 | 4 | 40.45 | 8 |  |  |  |  |  |
| **Microarray Cohort** | **CFS Mean** |  |  |  |  |  | 22.33 | 8.71 | 23.80 | 14.08 | 43.39 |
|  | **Control Mean** |  |  |  |  |  | 11.18 | 4.25 | 2.93 | 0.80 | 85.81 |
|  | **SD CFS** |  |  |  |  |  | 8.05 | 3.71 | 13.07 | 10.85 | 18.84 |
|  | **SD control** |  |  |  |  |  | 2.00 | 3.47 | 3.99 | 1.55 | 24.14 |
|  | **P-value** |  |  |  |  |  | **9.39E-05** | **9.44E-04** | **2.02E-05** | **9.50E-04** | **1.89E-07** |
| **Fractionated lymphocyte cohort** | **CFS Mean** |  |  |  |  |  | 23.2 | 9.6 | 23.2 | 14.4 | 41.3 |
|  | **Control Mean** |  |  |  |  |  | 11.0 | 2.9 | 3.4 | 2.4 | 87.0 |
|  | **SD CFS** |  |  |  |  |  | 6.6 | 4.5 | 13.3 | 7.9 | 12.8 |
|  | **SD control** |  |  |  |  |  | 2.1 | 1.4 | 4.0 | 3.1 | 9.5 |
|  | **P-value** |  |  |  |  |  | **3.15E-06** | **4.29E-05** | **4.42E-05** | **4.49E-05** | **1.07E-08** |
