# Supplementary material for: MicroRNAs hsa-miR-99b, hsa-miR-330, hsa-miR-126 and hsa-miR-30c: Potential Diagnostic Biomarkers in Natural Killer (NK) Cells of Patients with Chronic Fatigue Syndrome (CFS)/ Myalgic Encephalomyelitis (ME)
Source: PLoS One. 2016 Mar 11;11(3):e0150904. doi: 10.1371/journal.pone.0150904 (PMC4788442; doi:10.1371/journal.pone.0150904)
Supplement: S2 Table — (DOC) [file pone.0150904.s002.doc]

**Table S2.** 34 miRNA with differential expression between CFS/ME and matched controls; highlighting group size and means, fold change and P-values from Mann-Whitney U test before and after FDR correction.

| Gene | CFS (N.) | Control (N.) | Total (N.) | Fold Change | Mann-Whitney Exact Sig.(2-tailed) | FDR corrected P-value |
| --- | --- | --- | --- | --- | --- | --- |
| ambi_miR_7065 | 15 | 29 | 44 | 3.34 | 0.003 | 0.04 |
| hsa_miR_199a | 15 | 29 | 44 | 3.1 | 0.005 | 0.04 |
| hsa_miR_423 | 15 | 29 | 44 | 3.07 | 0.001 | 0.04 |
| ambi_miR_7058 | 15 | 29 | 44 | 3.05 | 0.0006 | 0.04 |
| hsa_miR_320 | 15 | 29 | 44 | 3.05 | 0.0006 | 0.04 |
| hsa_miR_27b | 15 | 28 | 43 | 3.03 | 0.007 | 0.04 |
| hsa_miR_145 | 15 | 28 | 43 | 3.01 | 0.009 | 0.04 |
| hsa_miR_339 | 15 | 28 | 43 | 2.99 | 0.005 | 0.04 |
| hsa_miR_185 | 15 | 29 | 44 | 2.93 | 0.007 | 0.04 |
| hsa_miR_191 | 15 | 29 | 44 | 2.91 | 0.003 | 0.04 |
| hsa_miR_30d | 15 | 29 | 44 | 2.89 | 0.003 | 0.04 |
| mmu_miR_140_AS | 15 | 29 | 44 | 2.88 | 0.003 | 0.04 |
| hsa_miR_27a | 15 | 29 | 44 | 2.88 | 0.006 | 0.04 |
| hsa_miR_181a | 15 | 29 | 44 | 2.88 | 0.007 | 0.04 |
| rno_miR_151_AS | 15 | 29 | 44 | 2.86 | 0.004 | 0.04 |
| mmu_miR_409 | 13 | 23 | 36 | 2.86 | 0.006 | 0.04 |
| hsa_miR_19b | 15 | 29 | 44 | 2.85 | 0.006 | 0.04 |
| hsa_miR_126 | 15 | 29 | 44 | 2.83 | 0.011 | 0.05 |
| hsa_miR_24 | 15 | 29 | 44 | 2.81 | 0.003 | 0.04 |
| hsa_miR_151 | 15 | 26 | 41 | 2.8 | 0.006 | 0.04 |
| hsa_let_7b | 15 | 29 | 44 | 2.78 | 0.007 | 0.04 |
| hsa_miR_30c | 15 | 29 | 44 | 2.78 | 0.007 | 0.04 |
| hsa_miR_92 | 15 | 29 | 44 | 2.76 | 0.006 | 0.04 |
| hsa_miR_103 | 15 | 29 | 44 | 2.73 | 0.006 | 0.04 |
| hsa_miR_99b | 15 | 23 | 38 | 2.66 | 0.003 | 0.04 |
| hsa_miR_22 | 15 | 28 | 43 | 2.59 | 0.007 | 0.04 |
| hsa_miR_326 | 15 | 22 | 37 | 2.47 | 0.002 | 0.04 |
| hsa_miR_422b | 15 | 28 | 43 | 2.3 | 0.008 | 0.04 |
| hsa_miR_197 | 15 | 25 | 40 | 2.29 | 0.004 | 0.04 |
| hsa_miR_210 | 13 | 23 | 36 | 2.22 | 0.008 | 0.04 |
| hsa_miR_324_3p | 15 | 24 | 39 | 2.2 | 0.004 | 0.04 |
| hsa_miR_331 | 15 | 24 | 39 | 2.07 | 0.01 | 0.05 |
| hsa_miR_324_5p | 9 | 14 | 23 | 1.92 | 0.009 | 0.04 |
| hsa_miR_330 | 10 | 14 | 24 | 1.58 | 0.007 | 0.04 |
