# Supplementary material for: MicroRNAs hsa-miR-99b, hsa-miR-330, hsa-miR-126 and hsa-miR-30c: Potential Diagnostic Biomarkers in Natural Killer (NK) Cells of Patients with Chronic Fatigue Syndrome (CFS)/ Myalgic Encephalomyelitis (ME)
Source: PLoS One. 2016 Mar 11;11(3):e0150904. doi: 10.1371/journal.pone.0150904 (PMC4788442; doi:10.1371/journal.pone.0150904)
Supplement: S3 Table — (DOC) [file pone.0150904.s003.doc]

**Table S3.** Genes demonstrating differential expression in primary NK cells after transfection with either pre-miR-99b or pre-miR-330-3P relative to a non-sense control transfection. Genes demonstrating fold changes >1.5 and P≤0.05 were selected for further analysis.

| **Symbol** | **Entrez_Gene_ID** | **Fold Change** | **P-value** | **Regulation** | **Transfected miRNA** |
| --- | --- | --- | --- | --- | --- |
| ABCA1 | 19 | 1.5 | 0.0026 | down | miR-99b |
| AKR1C4 | 1109 | 1.6 | 0.0305 | up | miR-99b |
| ALPP | 250 | 1.6 | 0.0383 | down | miR-99b |
| ARPC3 | 10094 | 1.6 | 0.0051 | up | miR-99b |
| ATG2A | 23130 | 1.5 | 0.0161 | down | miR-99b |
| C14orf85 | - | 1.5 | 0.0211 | down | miR-99b |
| C3orf34 | 84984 | 1.5 | 0.0221 | down | miR-99b |
| C7orf41 | 222166 | 1.6 | 0.0086 | down | miR-99b |
| CCT6A | 908 | 1.6 | 0.0029 | up | miR-99b |
| CD6 | 923 | 1.6 | 0.0015 | down | miR-99b |
| CTSL1 | 1514 | 1.8 | 0.0417 | down | miR-99b |
| DKK3 | 27122 | 1.7 | 0.0382 | down | miR-99b |
| FEZ1 | 9638 | 1.5 | 0.0359 | up | miR-99b |
| FLJ40722 | 285966 | 1.5 | 0.0044 | down | miR-99b |
| GZMB | 3002 | 1.6 | 0.0059 | up | miR-99b |
| HBA2 | 3040 | 1.6 | 0.0232 | up | miR-99b |
| Hs.567759 | - | 1.5 | 0.0098 | up | miR-99b |
| ICA1 | 3382 | 1.7 | 0.0168 | down | miR-99b |
| IFNG | 3458 | 2.6 | 0.0264 | up | miR-99b |
| IL17F | 112744 | 1.6 | 0.0160 | up | miR-99b |
| IL8 | 3576 | 1.5 | 0.0091 | down | miR-99b |
| KLRF1 | 51348 | 1.5 | 0.0308 | up | miR-99b |
| LOC644250 | 644250 | 1.6 | 0.0026 | down | miR-99b |
| MCM8 | 84515 | 1.6 | 0.0215 | down | miR-99b |
| MMP7 | 4316 | 1.7 | 0.0172 | up | miR-99b |
| NUBPL | 80224 | 1.7 | 0.0035 | down | miR-99b |
| PDCD4 | 27250 | 1.5 | 0.0132 | down | miR-99b |
| PMEPA1 | 56937 | 1.5 | 0.0183 | down | miR-99b |
| RPL39L | 116832 | 1.7 | 0.0013 | up | miR-99b |
| S100A4 | 6275 | 1.6 | 0.0389 | up | miR-99b |
| SGSM2 | 9905 | 1.5 | 0.0077 | down | miR-99b |
| STOM | 2040 | 1.6 | 0.0014 | up | miR-99b |
| THBS1 | 7057 | 1.6 | 0.0127 | down | miR-99b |
| TPM3P5 | 644330 | 1.5 | 0.0055 | up | miR-99b |
| TYMS | 7298 | 1.6 | 0.0012 | up | miR-99b |
| ULK1 | 8408 | 1.5 | 0.0300 | down | miR-99b |
| ZNF223 | 7766 | 1.5 | 0.0434 | down | miR-99b |
| **MMP9** | **4318** | **1.6** | **0.0373** | **down** | **miR-330-3P** |
| **SOD2** | **6648** | **1.6** | **0.0059** | **down** | **miR-330-3P** |
