# Supplementary material for: MicroRNAs hsa-miR-99b, hsa-miR-330, hsa-miR-126 and hsa-miR-30c: Potential Diagnostic Biomarkers in Natural Killer (NK) Cells of Patients with Chronic Fatigue Syndrome (CFS)/ Myalgic Encephalomyelitis (ME)
Source: PLoS One. 2016 Mar 11;11(3):e0150904. doi: 10.1371/journal.pone.0150904 (PMC4788442; doi:10.1371/journal.pone.0150904)
Supplement: S4 Table — Genes demonstrating fold changes >1.5 and P≤0.05 were selected for further analysis. (DOC) [file pone.0150904.s004.doc]

**Table S4.** Panther classification analysis of 39 genes deregulated by hsa-miR-99b and hsa-miR-330-3p in primary NK cells highlighting classification by biological process.

| **Biological Process** | **No. Classified genes** | **Deregulated Gene list** | **Expected** | **+/-** | **P value** | **Genes** |
| --- | --- | --- | --- | --- | --- | --- |
| cellular defence response | 457 | 5 | 0.8 | + | 1.16E-03 | IFNG; CD6; CTSL1; KLRF1; IL8 |
| macrophage activation | 305 | 4 | 0.54 | + | 1.97E-03 | IFNG; CD6; S100A4; IL8 |
| response to stimulus | 1798 | 9 | 3.16 | + | 3.20E-03 | IFNG; GZMB; THBS1; CD6; CTSL1; S100A4; HBA2; KLRF1; IL8 |
| immune system process | 2628 | 11 | 4.62 | + | 4.17E-03 | IFNG; GZMB; THBS1; CD6; CTSL1; S100A4; HBA2; KLRF1; IL8; SOD2; IL17F |
| oxygen and reactive oxygen species metabolic process | 63 | 2 | 0.11 | + | 5.56E-03 | SOD2; HBA2 |
| immune response | 756 | 5 | 1.33 | + | 9.90E-03 | IFNG; GZMB; S100A4; IL8; KLRF1 |
| response to interferon-gamma | 105 | 2 | 0.18 | + | 1.47E-02 | IFNG; IL8 |
| natural killer cell activation | 121 | 2 | 0.21 | + | 1.92E-02 | IFNG; KLRF1 |
| protein metabolic process | 3240 | 10 | 5.7 | + | 4.78E-02 | ULK1; MMP9; MMP7; GZMB; CD6; CTSL1; CCT6A; NUBPL; PDCD4; RPL39L |
| lipid transport | 246 | 2 | 0.43 | + | 6.95E-02 | ABCA1; HBA2 |
| nucleobase, nucleoside, nucleotide and nucleic acid metabolic process | 3825 | 3 | 6.72 | - | 7.49E-02 | TYMS; MCM8; ZNF223 |
| cell-cell signalling | 1331 | 5 | 2.34 | + | 8.15E-02 | IFNG; CD6; ICA1; IL17F; IL8 |
| negative regulation of apoptosis | 277 | 2 | 0.49 | + | 8.52E-02 | IFNG; CD6 |
| apoptosis | 966 | 4 | 1.7 | + | 8.80E-02 | IFNG; CD6; GZMB; PDCD4 |
| induction of apoptosis | 358 | 2 | 0.63 | + | 1.3E-01 | IFNG; PDCD4 |
| extracellular transport | 136 | 1 | 0.24 | + | 2.13E-01 | CD6 |
| metabolic process | 8267 | 17 | 14.53 | + | 2.48E-01 | MMP9; MMP7; GZMB: ABCA1; CTSL1; SOD2; TYMS; MCM8; CD6; ULK1; CCT6A; AKR1C4; HBA2; PDCD4; NUBPL; ZNF223; RPL39L |
| endocytosis | 575 | 2 | 1.01 | + | 2.68E-01 | CD6; KLRF1 |
